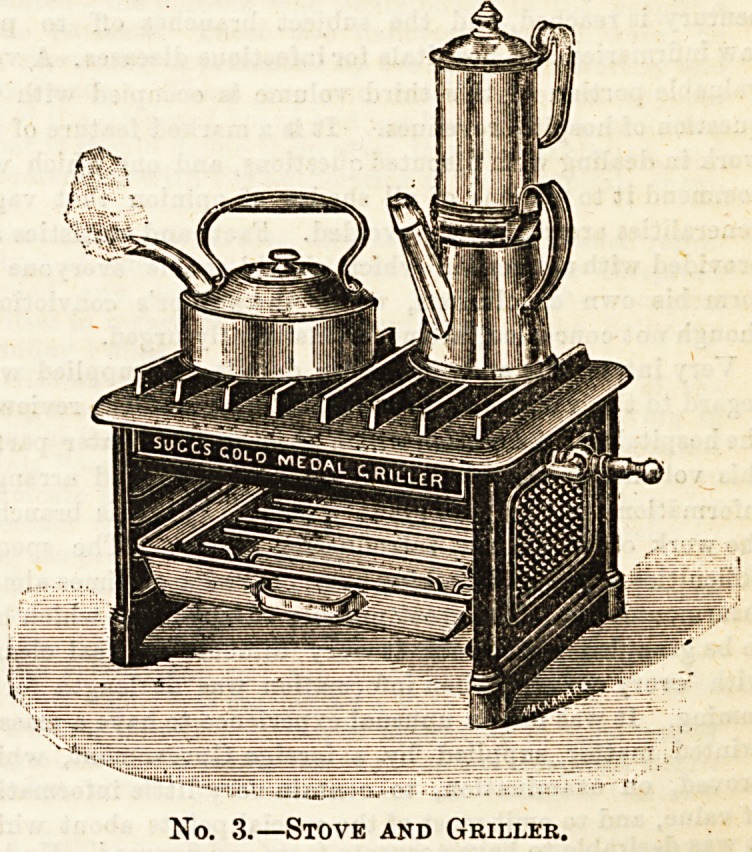# Cooking by Gas. IV

**Published:** 1893-01-28

**Authors:** 


### THE INSTITUTIONAL WORKSHOP.

### PRACTICAL DEPARTMENTS.

### COOKING BY GAS.—IV.

We have now discoursed on the applicability of gas for use in the kitchens of both public and private establishments. It but remains to enumerate its advantages for use by individuals and in the sick room. To none is gas of more value than to those who are dependent on the imperfect services to be obtained in apartments or chambers. The early morning breakfast through its use becomes a possibility under circumstances which would otherwise be insupportable. Individuals differ more in their requirements than do various establishments, but the obliging manufacturer is equally ready to supply any variety of tastes. If our aspirations do not soar higher than the making of tea or coffee, the simplest contrivance of all can be ours, and this without the service of the terrible plummer or expensive fittings. A simple indiarubber tube can be fixed by ourselves to our stove and thence to the gas-burner, whence we can procure our gas supply. Our first illustration shows this admirable little

No. 1.—Simple Stove.

arrangement, which, whether required for regular culinary use or not, will prove a most convenient possession; and it can be had for the small sum of 2s. It will be seen that it consists of a metal circle pierced at intervals, with fixtures above to act as a stand for the cooking utensil. The single row of tubing as shown above secures a very good heat, but double ones can always be obtained. Either are easy of

No. 2.—A Useful Stove.

complete regulation. If we desire to indulge in the luxury of hot milk with our coffee, Messrs. Sugg can offer us a more commodious stove, which affords standing-room for two utensils at the same time. The expense is small. The apparatus is shown in our second illustration. The room taken up by this stove is very little greater than required for the one we first described.

Should it fall to our lot to either provide the hot breakfast dish ourselves, or have to do without it, we can procure an excellent contrivance which supplies us with our grill, above which is a plate for the boiling apparatus. Thus provided, our bachelor establishment is most conveniently equipped. The price of this commodity is but 10s. 6d., and rather more when supplied with luminous burners. So small a sum is more than returned in the comfort gained. Only the most ambitious of amateur cooks will require any further cooking apparatus. If there are some, however, who desire to undertake all branches of cooking, "Sugg's Parisienne Roaster" will meet the requirements of the most exacting.

No. 3.—Stove and Griller.

It now only remains to point out the addition to the comfort of the sick room which the simple stove shown in our first illustration affords. It is infinitely preferable to the spirit-lamp usually resorted to in summer, and in winter it is much better than utilising the fire, as it is far more quietly manipulated. It can be adjusted in a few minutes, and used either on a bracket or table, conveniently situated, or conveyed to the fire-place. Thus in all departments of domestic economy to which gas can be applied for cooking purposes it reveals its utility; and should any be induced through us to try it themselves, we feel confident that they will coincide with us in giving it due praise.

## Figures and Tables

**No. 1. f1:**
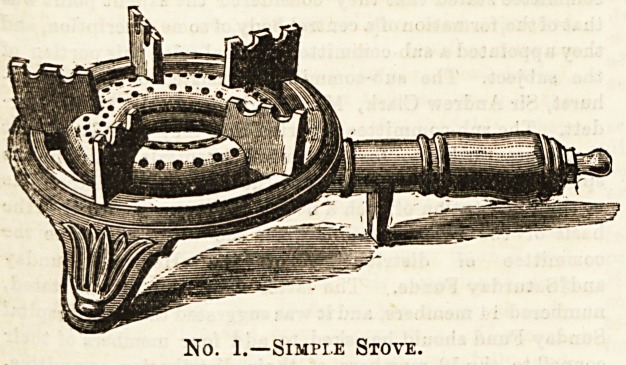


**No. 2. f2:**
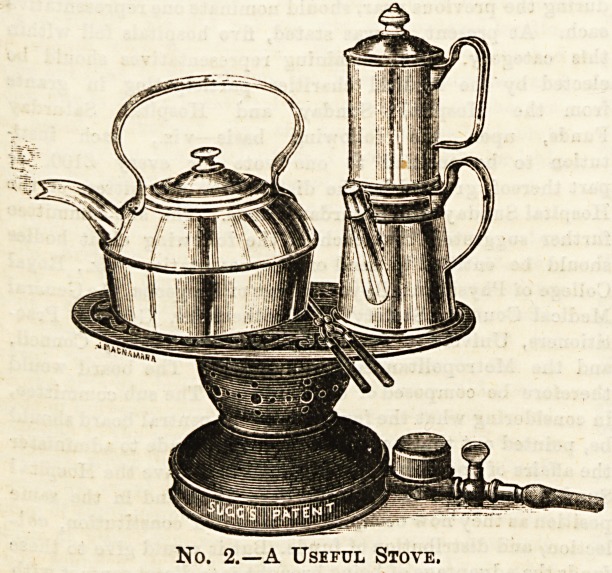


**No. 3. f3:**